# Acknowledgment to the Reviewers of *Viruses* in 2022

**DOI:** 10.3390/v15020278

**Published:** 2023-01-18

**Authors:** Viruses Editorial Office

**Affiliations:** MDPI AG, St. Alban-Anlage 66, 4052 Basel, Switzerland

High-quality academic publishing is built on rigorous peer review. *Viruses* was able to uphold its high standards for published papers due to the outstanding efforts of our reviewers. Thanks to the efforts of our reviewers in 2022, the median time to first decision was 16 days and the median time to publication was 38 days. Regardless of whether the articles they examined were ultimately published, the editors would like to express their appreciation and thank the following reviewers for the time and dedication that they have shown *Viruses*:
A.N. AnoopkumarKuldeep DhamaAaron BraultKuldeep GuptaAaron E. LinKumiko YoshimatsuAarthi NarayananKumiya SugiyamaAartjan J. W. te VelthuisKun YangAbdelaziz AbdelaalKun ZhangAbdul Kader JailaniKunihiko UmekitaAbdulaziz AlhazmiKurt E. WilliamsonAbdul-Cader Mohamed SarjoonKurt GustinAbdullah Sami SaribaşKurt ToblerAbel Viejo-BorbollaKushagra BansalAbelardo Silva-JúniorKushol GuptaAbhinay GontuKwang-Nyeong LeeAbhishek MishraKwong-Ming KeeAbuzer AliKyle RosenkeAchuit K. Singh Kylene Kehn-HallAd VosKyoko Tsukiyama-KoharaAda YanKyousuke KobayashiAdam BrufskyKyrkou IfigeneiaAdam M. SpivakLabrini V. AthanasiouAdam SchrumLaetitia CaniniAdam W. WhisnantLaia Bosch-CamósAdam WaickmanLakha SalaipethAdam ZlotnickLakshmi Sumitra VijayachandranAdarsh K. GuptaLambros PapayiannisAdebowale AdebiyiLance PresserAdelaide AlmeidaLarisa KarpenkoAdelaide MilaniLars RedeckeAdele Caterino-de-AraujoLászló EgyedAdeline E. WilliamsLászló ŐrfiAdhimoolam KarthikeyanLászló PalkovicsAdi BeharLászló SzilákAditya SharmaLaura Ambra NicoliniAdly Mohamed M. Abd-AllaLaura BerettaAdnan Mohsin AbdulazeezLaura GibsonAdolfo PomaLaura HertelAdrian OoLaura L. NewcombAdrian WhitehouseLaura LentiniAdriana CalderaroLaura Maria de PlanoAdriana ForeroLaura Medina-PucheAdriana GiriLaura PetrarcaAdriána LiptákováLaura PorrettiAdriana Patricia Corredor-FigueroaLaura SaderiAdriana VisonàLaura SicheroAdrianne JennerLaura TomassoneAdriano CarrascoLauren A. SiltzAdrien BreimanLaurent AndréolettiAgata StanekLaurent BigarréAgieshkumar Balakrishna PillaiLaurent Chatel-ChaixAgnieszka AdamekLaurent GilletAgnieszka K. SzczepankowskaLaurent MaillyAgnieszka ŁątkaLauro Velazquez-SalinasAgnieszka LembasLavanya ChallagundlaAgnieszka PawełczykLavínia Schüler FacciniAgnieszka Rynda-AppleLawrence M. WeissAgostino OgnibeneLawrence ShapiroAgus SunartoLe WangAhmad Jawad SabirLea HošnjakAhmed A. Al-KarmalawyLéa JoffrinAhmed A. ElhenawyLeah C. KatzelnickAhmed A. QuadeerLeandro Luongo MatosAhmed AliLechuang ChenAhmed ElbestawyLee CohnstaedtAhmed HadididLee Lee TengAhmed KandeilLee MakowskiAhmed Mostafa ElsayedLee SherryAhmed MoustafaLee W. GoneauAhmed Neamat-AllahLee-Jene LaiAhmed O. ShalashLei XuAhmed SayedLei ZhouAhmed TabbabiLeijian YongAhmed YousefLeike ZhangAileen RowanLeila Sabrina UllmannAiping WuLemonia SkouraÁislan de C. VivariniLeny JoseAitor NogalesLéo UchidaAjani AthukoralaLeonardo S. SantosAjay Kumar MishraLeonardo Silva BoiteuxAjit JagtapLeonardo VelascoAjitanuj RattanLeonid ChindelevitchAkbar DastjerdiLeonidas LotosAkira GotoLeonie UnterholznerAkira NakanishiLeontina BanicaAkira NishizonoLeopoldo PalmaÁkos BorosLeslie OgorzalyÁkos GellértLeslie ParentAlain VanderplaschenLeszek TylickiAlan BarrettLeticia Ruiz-GarciaAlan G. GoodmanLetizia Corinna MorlacchiAlan ParkerLia van der HoekAlan ReinLiana A. BasovaAlan WinstonLiana PlesAlba Fresco-Taboada Lianbing LinAlba Pérez-CataluñaLíbia Zé-ZéAlban RametteLibiao ZhangAlbert HeimLibo HeAlbert ZimmermannLidia Szulc-DąbrowskaAlberto BorghettiLidwien SmitAlberto Cedro-TandaLihong LiuAlberto ZanellaLihua WangAlbie Van DijkLijuan LiAldo MarroneLijun RongAlejandro ValbuenaLi-Kwan ChangAlejandro VallejoLiming LiAleksandr N. IshmatovLin WeiAleksandra Obrępalska-StęplowskaLina BaranauskieneAleksei A. StepanenkoLinbo ZhaoAlessandra BorsettiLinda BrunotteAlessandra Marnie Martins Gomes de CastroLinda DixonAlessandra SinopoliLinda HiebertAlessandro BartoloniLinda HouhamdiAlessandro GranitoLinda M. WakimAlessandro MalobertiLindell K. WeaverAlessandro MarcelloLinfa WangAlessandro PerrellaLing DengAlessandro PezzellaLing JinAlessandro SinigagliaLing QingAlessio BocediLing YueAlex ComptonLingling MiaoAlex Junior Souza de SouzaLionel FrangeulAlex M. MurphyLisa A. WaidnerAlexander Batista-DuharteLisa E. Hensley Alexander BelloLisa LindesmithAlexander BolshoyLisa MillerAlexander BorodavkaLisanework AyalewAlexander BukreyevLitao LiuAlexander ByrneLiubov KozlovskayaAlexander Crits-ChristophLiv BodeAlexander J. McMenaminLivia DonaireAlexander J. SzubertLivleen ShuklaAlexander KarasevLjubo BarbićAlexander M. ShestopalovLluís ColomoAlexander MottLogan BanadygaAlexander PakLogan W.F. DonaldsonAlexander PostelLois E. GreeneAlexander ProbstLokendra V. ChauhanAlexander SafatovLong SuiAlexander TarrLonnie van ZylAlexander TitzLoredana AlessioAlexander TonevitskyLoredana NicolettiAlexander V. ProninLoreé C. HellerAlexander Yurievich AlekseevLorenz LeitnerAlexander ZhgunLorenzo CapucciAlexandra BuckleyLorenzo FraileAlexandra GambaryanLorenzo LeonciniAlexandra J. MalbonLorenzo PaceAlexandra TauzinLorenzo PiermatteoAlexandre FabreLoreta KondiliAlexandre HassaninLori Emert-SedlakAlexandre LeitãoLoris RonconAlexandre MachadoLouie Mar A. GangcuangcoAlexandre OrsatoLouis AltamuraAlexandre Pérez-GonzálezLouis M. ManskyAlexandre StewartLouise CosbyAlexandru Florin RogobeteLouise PittAlexandru MovilaLouis-Marie BloyetAlexey SarapultsevLoukas ChatzisAlexis GalanisLourdes LledóAlfio GrilloLovorka BrajkovićAlfredo CastelloLubomira Nikolaeva-GlombAlfredo Diaz-LaraLuca CegolonAlfredo HernandezLuca CostanzoAlfredo TagarroLuca D. BertzbachAlgimantas PaulauskasLuca Danilo BertzbachAlhammad YousefLuca de SabatoAli MazloumLuca FerrariAlian LicoppeLuca FerrettiAlice ChateauLuca GallelliAlice Chiara ManettiLuca MarchettiAlicia Martínez-VareaLuca NervaAlicia Ponte-SucreLuca ScapoliAlicja WegrzynLuca Tudor GiurgeaAlicja Wiercińska-DrapałoLuca VangelistaAline Maria da SilvaLuca ZinzulaAlison BurmanLucía FernándezAllan ProchazkaLucía García-San MiguelAllan Randrup ThomsenLucia PintilieAllison DidychukLucia SignoriniAllison JohnsonLucia StanciakovaAlri PretoriusLuciana Barros de ArrudaAltaira D. DearbornLuciana CostaAlteri ClaudiaLuciana GalettoÁlvaro Francisco Lopes de SousaLuciano PironeAlvaro MallagarayLucie JašíkováÁlvaro Tamayo-VelascoLucio LuzzattoAmali SamarasingheLucrezia Ginevra LulliAmanda Carroll-PortilloLudmila V. PuchkovaAmanda DuffusLuigi BertolottiAmanda FecuryLuigi CeruloAmanda HannaLuigi MandrichAmar ParvateLuís Carlos de Souza FerreiraAmaresh DasLuis EnjuanesAmbar Lopez-MacayLuis F. NuñezAmbikapathi NivethaLuis Felipe Jave SuarezAmbrish SinghLuis Fernando MachadoAmelia Cervera OlagüeLuís Filipe MucciAmelia K. PintoLuis M. BrancoAmélie GuihotLuis Martinez-SobridoAmir GhorbaniLuis Menéndez-AriasAmir Sasan Mozaffari NejadLuis Perez Garcia-EstañAmir ShamshirianLuis ToroAmir SteinmanLuisa Salazar-VizcayaAmir-Babak Sioofy-KhojineLuiz Carlos KreutzAmit GoelLuiz CarneiroAmit KumarLuiza MendoncaAmr GamilLukas HegerAmr NegmLukas WeseslindtnerAmresh PrakashŁukasz ŚwiątekAmrita SarkarLung-Yi MakAmulya YaparlaLutz GöhringAmy B. RosenfeldLuuk. B. HilbrandsAmy GillgrassLuwanika MleraAmy H. DelgadoLuz Martín-CarboneroAmy SchuhLuzia Helena QueirózAmy StrydomLuzia Maria de Oliveira PintoAmy TangLynn DustinAmy Y. VittorLynn El HaddadAna Belen BlazquezLynnette C. GoatleyAna Belen Ruiz-GarciaM. C. GjessingAna C. CalpenaM. Joan CurcioAna Carolina Bernardes TerzianMa LuoAna CarvajalMaarit SuomalainenAna Claudia FrancoMaciej FrantAna DomenechMaciej ŻaczekAna DuarteMadeleine TråvénAna KlobucarMadhuri SubbiahAna Lorena Gutiérrez-EscolanoMadhuvanthi VijayanAna Maria Rivas-EstillaMadoka NakaiAna Paula Sayuri SatoMagali GarciaAna Pavla GurgelMagaly MartinezAna Raquel SoaresMagdalena BaymakovaAna ReisMagdalena DunowskaAna Rita Gonçalves CabecinhasMagdalena HurkaczAna SilvaMagdalena KrintusAna VučurovićMagdalena Makarska-BialokozAna-Belen BlazquezMagdalena Paplińska-GorycaAnan JongkaewwattanaMagile FonsecaAnanda Ayyappan Jaguva VasudevanMagnus EssandAnanda VasudevanMagnus ÖgrenAnass BouchnitaMahmoud A. SenousyAnastasia KotanidouMahmoud E. KhalifaAnastasia SveshnikovaMahmoud IbrahimAnastasia V. KomarovaMahmoud Mohamed NaguibAnatol PanasiukMahmoud Mohamed TawfickAnatoly N. KanatnikovMahmoud Mostafa AzzamAnatoly V. ZherdevMahmoud NaguibAnaya Edith RojasMahmoud SabraAnca DoceaMahmoud ShehataAncy JosephMahmut SafakAnda BăicușMai IzumidaAndanappa GadadMaite AubryAnders BergqvistMaite VaslinAnders BoydMaitreyi ShivkumarAndi KrumbholzMaja CupicAndre KochMaja M. LunarAndré SchreiberMaja ŠantakAndre Schutzer GodoyMakoto MakishimaAndrea BalliniMakoto OzawaAndrea BuscaroliMakoto UjikeAndrea CimarelliMakoto YamagishiAndrea DalbeniMaksim Leonidovich FilipenkoAndrea de VitoMakutiro MasavuliAndrea Di GioacchinoMalachy Ifeanyi OkekeAndrea FabbriMalcolm McConvilleAndrea GalliMałgorzata BlatkiewiczAndrea KroekerMałgorzata D. Klimowicz-BodysAndrea KrögerMalgorzata ŁobockaAndrea MagriMałgorzata Polz-DacewiczAndrea MarinoMałgorzata Pomorska-MólAndrea PalombieriMalik AydinAndrea Thoma-KressMalik SallamAndrea TrevisanMalin Flodström-TullbergAndrea Vukic DugacMamta Chawla SarkarAndrea ZalianiMana RaoAndreas E. VoloudakisManabu IgarashiAndreas JekleManasi TamhankarAndreas KuhnMandy MullerAndreas LindManel Essaidi-LaziosiAndreas SchlundtManickam AshokkumarAndreas SuhrbierManish DhawanAndrei A. DeviatkinManish Vijaya KumarAndrei Gheorghe MotocManmeet SinghAndrés Velasco-VillaManoj KumarAndrew D. HaddowManon NayracAndrew D. MillardManoochehr MakvandiAndrew HayhurstMansi SrivastavaAndrew J. HendersonManu AnantpadmaAndrew James BroadbentManuel BorcaAndrew MillardManuel GarrosaAndrew N. BubakManuel Gómez del MoralAndrew NorrisManuel MadrazoAndrew PetersManuela AntonioliAndrew R. CrowleyManuela CeccarelliAndrew ShawManuela DonalisioAndrew VarbleManuela OliveiraAndrew WargoManuela RizziAndrey CherstvyManuela SironiAndrey GoginManzoor RatherAndrey GrigorievMaolin LuAndrey LetarovMarc FuchsAndrey SolovyevMarc JaminAndrii SlonchakMarc JohnsonAndris KazaksMarc KottmaierAndriy KovalenkoMarc KvansakulAndrzej LangeMarcela LizanoAndrzej ZielezinskiMarceline CôtéAneela Zameer DurraniMarcella BassettoAneta RadziwonMarcello CandelliAneta SkaradzińskaMarcello CovinoAneta WoźniakMarcello LanariAngela BisioMarcelo AlmeidaAngela LoizidouMarcin OsuchowskiAngela M. Bosco-LauthMárcio Roberto Teixera NunesAngela PearsonMarco Aurélio Pereira HortaAngela R. BrancheMarco BinderAngela StufanoMarco Bruno Luigi RocchiAngelantonio MinafraMarco CiottiAngelika K. LootsMarco IannettaAngeliki KatsafadouMarco MontaltiAngeline RouersMarco RomitoAngelo PavesiMarco ThomasAngkana T. HuangMarco ZuinAngus WilsonMarcone Augusto Leal de OliveiraAniello MaieseMarcos Cesar GonçalvesAnil K. GiriMarcos Veiga dos SantosAnil KumarMarcus KaulAnisha Mahendra ThankiMarek PostulaAnissa ChouikhaMarek RadkowskiAnita HoweMarek WideraAnita K. McElroyMaren SchubertAnita M. SheteMargaret Adele ScullAnita ManogaraMargarida DuarteAnitha D. JayaprakashMargarita K. LayAnja de WeggheleireMargarita Martín CastilloAnja KiparMargo BrintonAnja KolaričMargus VarjakAnkana KakotiMaría A. AyllónAnke HuckriedeMaria Alessandra de MarcoAnke Renate Maria KraftMaría Belén PisanoAnke SchwarzMaria C. AlonsoAnn E. SluderMaria CapobianchiAnn Marie KimballMaria Carmen AlonsoAnn TollefsonMaria Cássia Jacintho Mendes CorrêaAnna BaraniakMaria Cecilia Garibaldi MarcondesAnna BellizziMaria Cristina PetraliaAnna BiernasiukMaria Daniela SilvaAnna Boroń-KaczmarskaMaría de Los A. Romero‐TlaloliniAnna HeitmannMaría del Carmen Valls MartínezAnna HymosMaría del Rosario Morales EspinosaAnna JackovaMaria DolciAnna Karolina MatczukMaria Elena MarcocciAnna KramvisMaría Eugenia GonzálezAnna LacastaMaria Eugenia Morales-BetoulleAnna MajewskaMaria ExindariAnna Maria PeriMaria Fernanda GrassiAnna MasiakMaria Fernanda PascuttiAnna OrłowskaMaria Florencia QuirogaAnna PiekarskaMaria Francesca CorteseAnna PikulaMaria Gabriela NovalAnna Rodolfa MalacridaMaria Gabriella Donà Anna SalvettiMaria Giuseppa SulloAnna ShtroMaria Grazia RomanelliAnna SomininaMaria Irene BelliniAnna TaglientiMaria JenckelAnna ToffanMaria João AlvesAnnamaria PratelliMaria K. DahleAnne A. GershonMaria Laura IddaAnne PohlmannMaria Leticia CruzAnne Sally DavisMaria Luisa MarenzoniAnnegret Pelchen-MatthewsMaria MazzitelliAnnelie PlentzMaria NevotAnnemarie E. LaumaeaMaria Nikolaevna TutukinaAnnette AudigéMaria P. BonasoniAnnibale PucaMaria PaparoupaAnnika E. MichelsenMaria PereiraAnthony E. KincaidMaria PironAnthony MarriottMaria Rios GrassiAnthony SchmittMaría Rosa BonoAntoine DurrbachMaria Serena BeatoAntoinette van der KuylMaria SzargutAntonella FrassanitoMaria T. SanchezAntonella OliveroMaria TempestaAntoni Soriano-ArandesMaria Teresa GiraudoAntonietta CuratolaMaria TokuyamaAntonino CataraMariah HassertAntonino PolimenoMariana MarinAntonio C. P. WongMariana Sequetin CunhaAntonio CascioMarianna KarachaliouAntonio CassoneMarianna T.P. FavaroAntonio Gregorio Dias JuniorMarianne Leruez-VilleAntonio MasMariano CarossinoAntonio MastinoMariano Nicolás BelaichAntonio Real-HohnMariano Pérez-FilgueiraAntónio RoldãoMariantonietta Di StefanoAntonio SantanielloMariarosaria CampiseAntonio Sarría SantameraMarie C. PizzornoAntonio TiberiniMarie Christine CadierguesAnuj AhujaMarie GallouxAnuj KumarMarie L. NguyenAnukumar BalakrishnanMarie PizzornoAnúp PandíțhMarie-Line AndreolaAnupriya AggarwalMarie-Theres PohlApostolos BeloukasMariette DucatezAravinthkumar JayabalanMariette F. DucatezArchana MishraMarijana StojanovicArgelia CuencaMarike GeldenhuysArghavan AlisoltaniMarina BobkovaArianna CalistriMarina BokAriel IsaacsMarina PadkinaArif SiddiquiMarina PapaianniArifa KhanMarina PotestaAriful IslamMarina SteinArmando Hernández GarcíaMarina StrakhovskayaArmando Mario DamianiMarine WasniewskiArmandodoriano BiancoMarino ParoliArmelle MaraisMario BrumArnab NayakMario CorbellinoArnaud G. BlouinMario EstableArne Broch BrantsæterMário Guimarães PessoaArnold GrünwellerMario I. OrtizArnold RabsonMario MietzschAron LukacherMario PerkovicArong GaowaMariola DutkiewiczArran J. FollyMarion DesdouitsArtem MetlinMarion HoggArtemis RumbouMarion LussignolArthur GruberMarion MigueresArthur KocherMarisa GranatoArturo Edgar Zenteno-GalindoMaritza N. Puray-ChavezArun KhattriMarius MasiulisArun Saravanakumar AnnamalaiMariusz MadejAruna AmarasingheMariyana GozmanovaArunava RoyMark Andrew WhiteArup BanerjeeMark Bodman-SmithAsami NishikoriMark BurkeAshaq AliMark D. HicarAshley BennettMark KrystalAshley OttoMark PeeplesAshwanth Christopher FrancisMark S. JohnsonAshwin RameshMark SlifkaAsisa VolzMark SuterAsiya Kamber ZaidiMark Thomas AndersenAsma Hatoum-AslanMark W. JackwoodAssefaw GebremedhinMark WillcoxAssia EljaafariMarko KutlešaAssunta BertacciniMarkus AntwerpenAstri Nur FaizahMarkus GlätzelAtashi SharmaMarkus HoffmannAtchara PhumeeMarkus M. KnodelAthanasia PavlopoulouMarkus ScholzÁtila RossiMarla Karine AmaranteAtsushi YamanakaMarlene DreuxAttila D. SándorMarta CanutiAttila FarsangMarta Coimbra MarquesAttila L. ÁdámMarta Denel-BobrowskaAttila TóthMarta GiovanettiAtze T. DasMarta Kuźmińska-BajorAude JaryMarta L. DeDiegoAudray K. HarrisMarta LuigiAudrey EsclatineMarta MassanellaAudrey Ferrier-RembertMarta PingarilhoAudrey LacroixMarta Sena VelezAugustin Mouinga-OndéméMartha A. Alexander-MillerAung KyawMartha BrownAurelie RakotondrafaraMartha OliveraAurora ParodiMartin BartasAurore RozièresMartin ChanAusra DomanskaMartin DorfAusrine AreskeviciuteMartin HölzerAusten L. TerwilligerMartin HufbauerAvinash PremrajMartin RafteryAvinash WaghrayMartin RichterAvneesh GautamMartin WelkerAwad A. ShehataMartina BergantAyako MiyazakiMartina KochAyça Arzu SayınerMartina SalákováAykut OzkulMartina SmolićAyman AhmedMaruti Jayram DhanavadeAyman MahmoudMarvin EdeasAysegul NalcaMarvin GrubmanAyumi SugiuraMarwa ElgendyAyushi RaiMary Anne TrabaudAzumi IshizakiMary F. KearneyAzwidowi LukhwareniMary HummelBaca ChanMary Lea KillianBaki AkgülMary WarrellBala ChandranMaryline SanterreBalaji ManicassamyMary-Louise PenrithBalal SadeghiMaryna StasevychBalázs FelföldiMarzia Puccioni-SohlerBálint KintsesMasaharu IwasakiBanhi BiswasMasahiro NiikuraBaochao FanMasahito NakanoBarbara BauceMasaji MaseBarbara BertasiMasako ShimamuraBarbara Di MartinoMasamichi MuramatsuBarbara KluppMasamichi NishiguchiBarbara MüllerMasanori AtsukawaBarbara RajtarMasanori MatsuiBarbara SchnierleMasashi NinomiyaBárbara Vieira do LagoMasayuki SaijoBarbie K. Ganser-PornillosMashael Al-NamaehBarry MarguliesMasmudur M. RahmanBarry MilavetzMassimiliano BergalloBarry RockxMassimo AmadoriBart RijndersMassimo CiccozziBartłomiej GrygorcewiczMassimo ColomboBasem A. AhmedMassimo FranchiniBasema SaddikMassimo La RosaBasudev PaudyalMassimo PizzatoBaudouin StandaertMassimo SpedicatoBeata Galińska-SkokMateja Manček-KeberBeata HalassyMateusz JankowskiBeata Hasiów-JaroszewskaMathias MartinsBeata Hukowska-SzematowiczMathias SchemmererBeate M. CrossleyMathieu DubéBeate M. KümmererMathieu IampietroBeatrice GraflMathieu MateoBeatrice MacchiMathieu PerreauBeatrice MercorelliMatías CastellsBeatriz ÁlvarezMatloob HusainBehboudi ShahriarMats LeifelsBelinda Baquero-PerezMatt JohansenBenedikt KauferMatteo BolcatoBenhur LeeMatteo CastelliBenjamin DewalsMatteo LegnardiBenjamin J. BurwitzMatteo NegroniBenjamin J. EckhardtMatteo RiccòBenjamin KimbleMatthew CollinsBenjamin MaasoumyMatthew D. WeitzmanBenjamin TrinitéMatthew TaylorBenjamin W. NeumanMatthew WeitzmanBenoit CallendretMatthew WiebeBenoit RemenantMatthias DobbelsteinBenoit VisseauxMatthias SchweizerBentley FaneMatthijs RaadsenBergmann Morais RibeiroMatti WarisBerislav BosnjakMattia CalzolariBernadette van den HoogenMattia MoriBernard J. HoetMaulik D. BadmaliaBernard KaićMaura DandriBernard LaganeMaureen LongBernard MossMauricio Comas-GarciaBernardo VegaMauricio García MateuBernhard KratzerMaurício Menegatti RigoBettie VoordouwMaurilia MarcacciBharat Bhusan PatnaikMaurizio NordioBharat MadanMauro Cesar Palmeira VilarBianca ZecchinMauro ViganòBilal AhmadMax CiarletBillekallu Thammegowda Naveen KumarMax Von KleistBin HeMaxime PichonBin LiMaxime WeryBin YuMaya ShmulevitzBinhua LiangMayara F. MaggioliBirke TewsMayke LeggewieBirte KalveramMazahar MoinBiswajit DasMd Bashir UddinBlake BallMd. Hakimul HaqueBlanca TaboadaMd. Imtaiyaz HassanBlessing Akombi-InyangMd. Iqbal HossainBluma G. BrennerMd. Jamal UddinBo LiangMd. Jashim UddinBo NiklassonMeaghan HancockBo PengMee Seon KimBo ZhangMegan SteainBoaz ArziMehmet OzaslanBobo MokMehmet Şengün ÖzsözBojan MitićMei X. WuBonnie J. LafleurMeichen PanBoris M. HogemaMei-Ling LiBoris RevolloMelania M. AmorimBoyan GrigorovMelina FischerBo-Young HongMelinda Jenkins-MooreBrad S. PickeringMelissa Bello-PerezBrandon J. BeddingfieldMelissa MaginnisBrandon ReynekeMengxian LongBranka MarinovicMenira SouzaBratko FilipičMeritxell García-QuintanillaBreck A. DuerkopMich MuldersBrendan BellMichael A. PurdyBrent A. StanfieldMichael B. TownsendBrent CredilleMichael BukrinskyBrian BothnerMichael BuschBrian GowenMichael ConwayBrian JordanMichael D. PurdyBrian LadmanMichael EllerBrian MurphyMichael G. IsonBrian SalmonsMichael GershonBrian WigdahlMichael GlickBritta MöhlMichael H. PetersBruce BanfieldMichael HessBrunno CaetanoMichael HolbrookBruno HernaezMichael KulkaBryce M. WarnerMichael LetkoBrygida KnyszMichael LoBurton F. DickeyMichael M. C. LaiByung-Hoon JeongMichael M. ThomsonC. Joaquin CaceresMichael McGowanC. Sabrina TanMichael McVoyC. Thomas BockMichael MerchlinskyCaijun SunMichael MintsCaitlin A. O’BrienMichael MuehlebachCamila Michele AppolinárioMichael P. ReichelCamilla MucciniMichael RiehleCamus ChristelleMichael S. PiepenbrinkCapai LisandruMichael SummersCara BrookMichael TiszaCarina ReuscherMichael VeitCarine MaisseMichaela RumlováCarl Jonas Jorge SpetzMichaela SchmidtkeCarl LavieMichal KajsíkCarla FontanaMichal LinialCarla PereiraMichel PépinCarlos A. BuenoMichel RavelonandroCarlos AriasMichele CameroCarlos BritesMichele Della BartolaCarlos Daniel Gornatti-ChurriaMichele LaiCarlos Eduardo Fonseca-AlvesMichele SpinicciCarlos H. RomeroMichele TonelliCarlos Horacio Acosta MuñizMichelle FlennikenCarlos MartinsMichelle J. GroomeCarlos Medicis MorelMichelle L. D’AntoniCarlos PrudencioMichelle Mendiola PlaCarlos Sandoval-JaimeMichelle MeyerCarlos ViegasMichelle N. MeyerCarmela PaolilloMichihiro TakagiCarmelo López del RincónMichikazu NakaiCarmen de MendozaMicholas SmithCarmen EmbregtsMiguel A. ArandaCarmen HernándezMiguel Angel Chávez-FumagalliCarmen Hidalgo-TenorioMiguel Angel Muñoz-AlíaCarmen Maria Gonzalez DomenechMiguel Ángel Rodríguez-MillaCarmen Nussbaum-KrammerMiguel JuárezCarmen Sílvia Valente BarbasMiguel Julián Martínez YoldiCarmine CoppolaMiguel López-FerberCarol Chitko-MckownMiguel MatosCarol D. WeissMiguel Ponce-VargasCarol L. SabourinMiguel Rodríguez-PulidoCarol StepienMihaela HostiucCarolina Colombelli PaccaMihaescu GrigoreCarolina RosadasMihai NitulescuCarolina RosasMihai OlteanCaroline CharreMihnea BostinaCaroline GilbertMikael SkurnikCarsten MunkMike FlintCaryn L. HeldtMikeljon NikolichCassandra KohMikhail A. DziadzkoCatalina FlorezMikhail Oliveira LeastroCatharine N. WelchMikhail Y. InyushinCatherine A. FreijeMilad ZandiCatherine EichwaldMilan DejmekCatherine InizanMilan MladenovicCatherine Vénien-BryanMilly Ming Ju ChoyCécile PhilippeMilton SubbsCecília de Souza ValenteMin FangCecilia M. EgoavilMin LangCecilia Söderberg-NauclerMin SunCelia AbolnikMin YangCelia BoukadidaMinetaro AritaCemal ÜnMing LuoCemil ColakMing Ming ZhouCesar EscalanteMing XiaCesar Marcial Escobedo-Bonilla Escobedo-BonillaMing YangCesare Ernesto Maria GruberMingchong YangChanakha NavaratnarajahMingchu ChengChandru SubramaniMinghang WangChang Gyu WooMingliang HeChang HuangMing-Tsan LiuChang-Chi LinMing-Wei LuChanghoon ParkMingxian ChangChang-Seon SongMing-Yeng LinChang-Tong YangMinh HoangChantal J. SnoeckMinhua LuoChantel SloanMinseon ParkChao GengMin-Sik KimChao WuMinze ZhangChao YeMira A. M. BehnamChao ZhangMiranda BertramChaozheng LiMiranda de GraafCharalampos KartsiosMiri KrupkinChari CohenMiroslav GlasaCharles BanghamMiroslav P. IlićCharles Brandon StauftMisa KorvaCharles CookMisako KonishiCharles H. CalisherMitsutoshi YoneyamaCharles LubelczykMizuho NitaCharles N. AgotiMizuki YamamotoCharles NfonMo SalmanCharles P. VendittiMogens KilstrupCharles ShoemakerMohamad-Hani TemsahCharles StauftMohamed AbouzidCharles W. ArmitageMohamed Ashour FikryCharlotte UetrechtMohamed El KassasCharu RajputMohamed El-MokhtarChaturaka RodrigoMohamed Ismail AshrafaliChe C. ColpittsMohamed SaidChee Wah TanMohammad AatifChen LiangMohammad Al-HatamlehChenglei LiMohammad HajizadehCheng-Lin WuMohammad HeidariCheng-Wen LinMohammad SadraeianChengwu ZouMohammad Ziaur RahmanCheng-Yao YangMohammadali Khan MirzaeiChenjian GuMohammed NooruzzamanChetan MeshramMohammed Salah Al-RadhiCheuk-Wai Horace ChoiMohan HaleyurgirisettyChi LiMohan MaheshChia-Chi WangMohanned Naif AlhussienChia-Chun TsengMohd Ikram AnsariChia-Chyi LiuMohd ImranChiao-Fang TengMohd RaufChiara ChiozziniMohie HaridyChiara CiprianiMohit ChawlaChiara PinoniMohsen KerkeniChiara PiubelliMohsen KeshavarzChia-Yi ChangMohsin JafriChichun HoMohube MaepaChien Hung ChenMoises Leon-JuarezChien-Hui HungMojgan RabieyChien-hung YuMomtchilo RussoChien-Ming ChaoMonali C. RahalkarChih-Yang WangMonica BassoChikara MasutaMonica BirkheadChin Ho ChenMónica J. RothChinmay V. TikheMonica Malone McNealChiu-Ming WenMonick Lindenmeyer GuimarãesChiwai KanMoniek de WitteChi-Young WangMonika PobiegaChou-Zen GiamMonika RinderChris L. SiepkerMonique van OersChris RobinsonMonique VogelChris VerschoorMonzurul RoniChristelle FabletMookkan PrabakaranChristelle Mbondji-WonjeMorgane SolisChristian A. DevauxMorihito TakitaChristian BarbatoMorteza YousefiChristian BrendelMostafa F. N. AbushahbaChristian CambillauMotoshi TajimaChristian GortázarMourad BendjennatChristian GrønhøjMoyasar AlhamoChristian H. IhlingMubarak S. KarsanyChristian NdekeziMudan ZhangChristian NusshagMudit TyagiChristian PfallerMuhammad Altaf KhanChristian SauderMuhammad AshfaqChristian SinzgerMuhammad Atif ZahoorChristiane Weissenbacher-LangMuhammad ImranChristiane WobusMuhammad IqhrammullahChristina CarlanderMuhammad KashifChristina L. GardnerMuhammad NomanChristina LeysonMuhammad Shah Nawaz-ul-RehmanChristina TsigalouMuhammad SohailChristine A. KingMukhlid YousifChristine CousensMunawar NaylaChristine E. EngelandMunir IqbalChristine PourcelMunywoki PatrickChristoph BuchtaMurali MunirajuChristoph KnosallaMuthu ThiruvengadamChristoph KreerMya Myat Ngwe TunChristoph SchultheißMyoung Kyu LeeChristoph WelschMyriam ErmonvalChristophe ChesneauMyrna M. MillerChristophe ChevalierNabab KhanChristophe PrehaudNader RahimiChristophe RodriguezNadia TouilChristopher AikenNadim AjamiChristopher Byron BrookeNadine KrügerChristopher E. DenesNaga Venkata Sabarinath NeerukondaChristopher Hamilton-WestNagaraj P. ShettiChristopher HammerbeckNagendra Nath BarmanChristopher HellenNagesh KolishettiChristopher J. DestacheNagib AhsanChristopher L. NethertonNajim ZaferChristopher LupferNan HaoChristopher PayanNanda Kishore RouthuChristopher R. M. AsquithNanjie DengChristopher S. AndersonNaoki IkegayaChristopher Wildrick WoodsNaoki OishiChrysoula G. OrfanidouNaoki TanakaChuan ChenNaoru KoizumiChun Kwok WongNaoto ItoChung-Chi HuNaoyoshi MaedaChung-Da YangNardus MollentzeChunhe GuoNarelle NancarrowChun-Ming LinNatalee NewtonChunyan ZhaoNatalia CheshenkoChunyang GuNatalia GolenderChun-Yu ChenNatalia KurhalukChutima ThepparitNatalia LauferCibulski SamuelNatalia LomakinaCigdem AlkanNatalia Mazur-PanasiukCihan YurdaydinNatalia OsnaCino-Ozuna A. GiselleNatalia RamosCinzia AuritiNatalia Soriano-SarabiaCiprian TomuleasaNatalie Dailey GarnesCiro Pasquale RomanoNatalie M. KirkClaire DebackNataša KnapClaire DeleageNatasha N. GaudreaultClaire I. PresneNatasha Tilston-LunelClaire PallesNatasza Borodynk-FilasClaire R. SharpNathalie BissonnetteClaire ThorneNathan CowiesonClare JollyNathan RothClarissa DamasoNathan ShererClaude RispeNathaniel DenkersClaudia CambreaNathaniel LandauClaudia Maria TucciaroneNathaniel MoormanClaudia MatteucciNauman AhmedClaudia PileggiNaveed AsgharClaudia RückertNaveen KumarClaudia Soledad SepúlvedaNeal A. DeLucaClaudia VanettiNeeltje KootstraClaudia VillicañaNeeltje van DoremalenClaudia WylezichNeil BerryClaudia ZanardelloNeil ChristensenClaudio de MartinisNeil D. ChristensenClaudio FeniziaNejat DüzgüneşClaudio W. CanalNelmary Hernandez-AlvaradoClayton WinklerNenad PandakClinton JonesNenavath Gopal NaikClinton R. PadenNeta ZuckermanColin ButtimerNewbrook KerryColin ParrishNghia P. TruongConcetta CastillettiNguyen T. K. VoConnor BamfordNiall P. ConlonConrad FreulingNicholas ChesarinoConstant GillotNicholas DopkinsConstantinos PetrovasNicholas F. ParrishCorinne Schmitt-KeichingerNicholas J. HaleyCornel BaltăNicholas J. LennemannCornelia HeinzeNicholas JohnsonCorneliu Petru PopescuNicholas KomarCosimo GallettiNicholas SvitekCosimo LupoNick Fountain-JonesCosmin CituNick JuleffCostanza di ChiaraNicla RomanoCostin Teodor StrebaNicola BartolomeoCouret JannelleNicola DecaroCraig BrunettiNicola F. FletcherCraig ForrestNicola MumoliCraig McCormickNicolae GicaCraig R. ChristieNicolas BejermanCraig RossNicolás GálvezCriscuolo ElenaNicolas LévêqueCristian BaicusNicolas RuggliCristiana GarciaNicolas SolerCristiana VoicuNicolas TremblayCristina CostaNicole BarpCristina Dopazo TaboadaNicole FischerCristina Ferrer-OrtaNicole N. HaeseCristina MarzachíNicole R. SextonCristina Pinedo-RivillaNicole VidalCristina RiscoNicoletta CantaruttiCristina RussoNicolina CapoluongoCristina W. CunhaNicolò MussoCristóbal ChaidezNidhi ShuklaCrystal W. BurkeNigel CookCsaba VízlerNikolaos MachairasCsabai ZsoltNikolaos SiafakasCui GuoNikolaos StilianakisCunbao LiuNikolas FriedrichCynthia A. Pise-MasisonNikon VassilakosCynthia Chester CardosoNilakshi BaruaCynthia DerdeynNils GassenCynthia J. Koziol-WhiteNils LannesCyril Le NouenNina Haagenrud SchultzCzeslaw RadziejewskiNina TikunovaDa DiNoa Berar-YanayDachrit NilubolNoam ErezDae-Gyun AhnNobuhiko OkabeDaesik ParkNobuko TunoDai WangNobumitsu SasakiDaihai HeNoelia Carmona-VicenteDainius ZieniusNoha Mousaad ElemamDalia Anwar HamzaNoman SiddiquiDalila CrucittiNomatter ChinganduDalius ButkauskasNora M. ChapmanDamien CoupeauNorbert J. RobertsDan HellerNorikazu IsodaDana VanlandinghamNorma SantosDaniel A. TruchadoNormand Garcia-HernandezDaniel Ardisson-AraújoNorsazilawati SaadDaniel BeckerNoula ShembadeDaniel C. NelsonNuredin HabiliDaniel CandottiNuria MontesDaniel CohenOana SăndulescuDaniel DepledgeOcean ThakaliDaniel DeredgeOgnjen PerišićDaniel DiMaioOhad MazorDaniel DuniaØivind BerghDaniel ElbirtOlaf IskenDaniel EllederOlafur SigurjonnsonDaniel Enosi TuipulotuOle Herman AmbúrDaniel GiglioOleg O. GlebovDaniel GómezOleg RyabchykovDaniel Gonçalves-CarneiroOleksandr NarykovDaniel J. FelmleeOleksandr ZinenkoDaniel JenkinOlga BelykhDaniel Marcelo CisternaOlga DolnikDaniel NobachOlga E. IvanovaDaniel NoyolaOlga I. YarovayaDaniel P. DepledgeOlga SokolovaDaniel RuzekOliver EalesDaniel SalamangoOliver HendricksDaniel SchmidtOlivier CassarDaniel ScottOlivier EscaffreDaniel TodtOlivier MarionDaniela BezemerOlivier MauffretDaniela ProverbioOlivier PiconeDaniela Toro-AscuyOlivier RestifDaniele ArmeniaOlivier RohrDaniele FocosiOlivier SchwartzDaniele LapaOlufemi Emmanuel BabalolaDanielle AndersonOlve PeersenDanielle R. AdneyOm Prakash ChoudharyDaniil IvanovOmar NyabiDanijela ČerneOmbretta TurrizianiDann TurnerOmkar Vijay ByadgiDante R. CulquiOmnia KutkatDanuta Januszkiewicz-LewandowskaOren KobilerDara A. LehmanOrkide KoyuncuDaria AugustyniakOrlando Torres-FernándezDaria MezhenskayaOsama A. KishtaDaria Mochly-RosenOsamu HottaDario CalafioreOsbourne QuayeDarko VončinaOscar A. NegreteDarren JardineOscar CastilloDarryl FalzaranoOscar CorderoDarwin A. León-FigueroaOscar D. SalomónDaryl LauOsman AktaşDat HaOtávio EspíndolaDavid A. BenfieldOthmar EngelhardtDavid A. LawrenceOttmar HerchenröderDavid A. MacLeodOumar TraoreDavid A. WohlOuti LyytinenDavid AlmenarOvidiu PopDavid BoutolleauOwais A. KhanDavid CourtneyPablo GastaminzaDavid D. FischerPablo Guardado-CalvoDavid D. L. MinhPablo Thomas-DupontDavid DuniganPakieli H. KaufusiDavid FernigPakorn AiewsakunDavid FrickPallabita ChowdhuryDavid GarfinkelPam Dachung LukaDavid HarleyPamela Di GiovanniDavid HawkesPamela NicholsonDavid HawmanPan TaoDavid HouPanagiotis D. KatsoulosDavid J. NolanPanagiotis PeitsidisDavid J. RowlandsPanayampalli Subbian SatheshkumarDavid J. SpiroPankaj PandeyDavid JuhlPankaj SharmaDavid KingsleyPaola Carolina Valenzuela-LeónDavid LukacPaola de BenedictisDavid M. KoellePaola del PortoDavid McVeyPaola FaverioDavid OrnellesPaola LondeiDavid PatonPaola QuaresimaDavid PeabodyPaola StoriciDavid PoloPaolo BoffanoDavid SchultzPaolo BonilauriDavid SteffenPaolo CalistriDavid T. HarrisPaolo CapozzaDavid TomatPaolo CarraroDavid van de VijverPaolo CastigliaDavid W. BeasleyPaolo FagoneDavid W. DyerPaolo MeneguzzoDavid WiesenfeldPaolo PastorinoDavide ColombiParmit SinghDavide SoglianiParul SharmaDawei ZhouParvaiz Ahmad NaikDa-Yuan ChenParvez Mahmood KhanDay-Yu ChaoPascal BittelDean D. ErdmanPascale Krejbich-TrototDebajit DeyPasquale AgostiDebashis DuttaPasquale SaldarelliDebby van RielPasquale VitoDebipreeta BhowmikPasqualina D’UrsiDeborah Jacobs-SeraPatricia Agudelo-RomeroDebra WadfordPatricia DayDeepak B. Thimiri Govinda RajPatricia GarcezDeepak Kumar YadavPatricia LeglerDefang ZhouPatricia PesaventoDefu YaoPatricia Recordon-PinsonDelia MunteanPatrick ArbuthnotDelia TitPatrick DemanaDemetra S. M. ChatzileontiadouPatrick HaDenis KainovPatrick HearingDenis KolbasovPatrick McClureDenis PiérardPatrizia CavadiniDennis GroganPatryk FrąckowiakDennis ÖzcelikPatryk TarkaDennis RubbenstrothPaul A. RotaDennis WrightPaul BertschDenys A. KhaperskyyPaul DabischDerek E. DimcheffPaul DenyDerek J. RoyerPaul G. HiggsDerseree ArcharyPaul HershbergerDer-Shan SunPaul HickDespoina BerisPaul KinchingtonDetlef E. DietrichPaul LehmannDetlev KrugerPaul R. GrantDevadas KrishnakumarPaul RotaDevendra Kumar RaiPaul SloweyDevika SirohiPaul W. BlairDevin A. BowesPaula F. ZamoraDevojit Kumar SarmaPaula Luize Camargos FonsecaDewald SchoemanPaula PristDharmendra Kumar YadavPaulo Dell’Armelina RochaDhawal ShahPaulo SanchesDhruv DesaiPaulraj KanmaniDi LiuPavel A. NazarovDiana J. M. van Den WollenbergPavel SolopovDiana MieliauskaitėPawan SinghDiana OrtizPaweł BęczkowskiDiane GriffinPawel BieganowskiDianjun CaoPawel ZmoraDianne LangfordPawin PadungtodDiego A. CaraballoPedro A PiedraDiego AllonsoPedro A. AraújoDiego AlvarezPedro Augusto AlvesDiego Carrillo-BeltránPedro Luis Ramos-GonzalezDiego E. AlvarezPedro PellicerDiego ForniPedro Plans-RubióDiego La MendolaPei XuDiego Leoni FrancoPei ZhouDiego Quito-AvilaPeifang SunDieter HoffmannPeiqi YinDilip K. NagPeirong JiaoDimitrios CassimosPeiwu QinDimitrios PapagiannisPenelope Kay-FedorovDinko NovoselPeng HeDinler AntunesPeng ZhouDiogo PratasPeng ZouDipayan BosePenghua WangDirk J. van LeeuwenPengxiang ChangDirk NettelbeckPenny ClarkeDirk WohlleberPere GodoyDiwaker TripathiPero LucinDmitriy ShcherbakovPerran RossDmitriy V. MazurovPerugu ShyamDmitry KireevPerumal RenukadeviDmitry KostyushevPeter A. DurrDmitry V. MukhaPeter A. IvanovDoina AtanasiuPeter A. MinchellaDolyce LowPeter BraunDomenico AcanforaPeter CherepanovDomenico Attilio RomanelloPeter DelputteDomenico BenvenutoPeter J. EggenhuizenDomenico FrezzaPeter J. WalkerDomenico GalantePeter JarcuskaDominic WichmannPeter Johannes HolstDominique L. ChaputPeter KirklandDominique SchollsPeter KrugDonald A. McFarlanePeter MedveczkyDonatella FiorePeter PalukaitisDongdong CaoPeter PelkaDonghoon ChungPeter PushkoDonglei SunPeter RudgeDonia BouzidPeter StäheliDonna A. MacDuffPetras PrakasDonna NeumannPetros RafailidisDorian McIlroyPhebe De HeusDorit NitzanPhilip El-DuahDorota KmiecPhilip M. MurphyDorota SikorskaPhilip MeulemanDorota Zarębska-MichalukPhilip NgDorothee KieferPhilip R. SpradlingDorothee Von LaerPhilip SerwerDouglas AdamoskiPhilipp A. IlinykhDouglas Bardini SilveiraPhilippe BrouquiDouglas C. HooperPhilippe ChouteauDouglas CauseyPhilippe DesprèsDouglas E. BrackneyPhilippe Le MercierDouglas E. VetterPhilippe RascleDouglas GladuePhillip C. GaugerDouglas J. LaCountPhillip S. CoburnDouglas LeamanPhilma Glora MuthurajDr. Jayanta MondalPhirom PrompiramDragan BrnićPhoebe Stevenson-LeggettDragomira MajhenPhuong Thi HoangDragos Catalin JianuPiergiuseppe de BerardinisDrishya DiwakerPierluca PiselliDu YuchunPiero ColombattoDuane P. GrandgenettPierre CappyDulce C.N. SantosPierre RoquesDuygu Sari-AkPierre-Emmanuel CeccaldiDwayne RoachPietro FerraraEain A. MurphyPietro Hiram GuzziEberhard HildtPilar Martin-DuqueEd GalyovPin-Huan YuEdeildo Ferreira Silva-JúniorPin-Nan ChengEdel Barbosa-StancioliPio ContiEdiane SilvaPiotr GolecEdith PorterPiotr GrabarczykEdmond J. RemarquePisano María BelénEduard Puente MassaguerPluvio CoronadoEduardo BerriatuaPo Gee Genevieve FungEduardo Gomez-CasadoPo Ying ChiaEduardo Montoya-DiazPo-Huang LiangEduardo OsunaPoliane ZerbiniEdvīns OļševskisPo-Lin ChiuEdward GershburgPonsiano OcamaEdward StephensPoonam MathurEdyta Kaczorek-ŁukowskaPorntippa LekcharoensukEdyta ŚwiętońPoulami SarkarEeva AuvinenPouya HassandarvishEeva TuppurainenPo-Yuan KeEfrem FogliaPrabu GnanasekaranEfrem LimPradeep D. UchilEggi ArguniPradhib VenkatesanEiichi KodamaPrakasha KempaiahEiji MatsuuraPramodh VallurupalliEike Roman HrinciusPranav ShahEiko MatsuoPrapansak SrisapoomeEileen RoulisPrasad ParadkarEkaterina KolesanovaPrasanth ManoharEkaterina StepanovaPrashant DesaiElana EhrlichPrashant SharmaElena A. ProkopyevaPrashanth Kumar Bhusetty NageshElena Cecilia RoşcaPratima KumariElena DelgadoPratyusha MandalElena GiorgiPravata K. PradhanElena GovorkovaPrerna DabralElena Lo PrestiPriscila M. S. CastanhaElena ParianiPriscilla TngElena PomariPritesh DesaiElena SavvateevaPriti Kumar RoyElena V. OrlovaPritom ChowdhuryElena-Mihaela CordeanuPrzemysław DecewiczEleni TzikaPuja KumariEleonora CellaQi ShenEleonora PonterioQian WangEliane MeursQiang LiuEliette BonnefoyQiang SunElina HoreftiQiaosheng PuElina LaantoQibin GengElio CenciQihan LiElisa VicenziQin ZhaoElisa ZanottoQinfang LiuElisabeta AntonescuQing PanElisabeth A. FalendyszQing ZhangElisabeth Puchhammer-StöcklQingbing ZhengElisabetta DegasperiQingmei XieElisabetta Di FeliceQingquan ChenElisabetta ManaresiQinxue HuElisheva SmithQiubai LiElizabeth LockhartQiwei QinElizabeth Natal De-GaspariQiwei ZhangElizabeth Ramirez-MedinaQiwen DongElizabeth RoyallQiya ZhangElizabeth V. FowlerQiyi TangElizabeth ZumbrunQuan ZhongElizaveta StarodubovaQuarraisha Abdool KarimElke BognerQuentin Lamy-BesnierElla MendelsonQuirina Santos-CostaEllen KrautkrämerQunying MaoElliott F. MiotRachael E. TarlintonEloise MastrangeloRachael PungElrashdy M. RedwanRachael TarlintonElsayed M. AbdelwhabRachel A. SattlerElvio LepriRachel PalinskiElvira Garza GonzálezRachel RoperElyes ZhiouaRachel Yoon Kyung ChangEman AnisRachele CaglianiEmil KupekRachy AbrahamEmilia HadziyannisRadha GopalEmilia JaskułaRadu BotezatuEmiliana BrocchiRafael Bayarri-OlmosEmilie PondevilleRafael Brandao VarellaEmilien JeannotRafael DelgadoEmilio ParisiniRafael FrancaEmily JohnRafael Jacinto VillanuevaEmilyn Emy MatsumuraRafael Rivera-BustamanteEmma L. MohrRafał Białynicki-BirulaEmma NilssonRaffaele de FrancescoÉmmanuel BréardRaffaele La PortaEmmanuel DrouetRagunathan DevendranEmmanuel KabweRaied Abou KubaaEmmanuelle BignonRaimo PohjanvirtaEmmanuelle MullerRainer UlrichEmmanuelle VigneRajagopal MuruganEnass A. Abdel-HameedRajagopalan SaranathanEnder DinçerRajagopalbabu SrinivasanEng Soo YapRajesh PandeyEngy ElekhnawyRajesh Ramachandra SinghEnikő FehérRajib DebEnric MateuRajiv N. ShahEnrica SozziRajiv PathakEnrico M. CamporesiRajkumar ChinnaduraiEnrico PiraRajneesh KumarEnzo Z. PoirierRalf BartenschlagerEphraim M. HanksRalf DietzgenEr YueRalf KircheisErez Bar-HaimRalf WagnerEric AgboliRaluca Ecaterina HaligaEric BortzRamendra Pati PandeyEric ChampagneRamin Raul Ossami SaidyEric D. SpitzerRamón AlemanyEric GoffinRamon GonzalezEric J. SchottRamona Gabriela UrsuEric JanRamona MöglingEric M. MuckerRana AbdelnabiEric MosselRana ChakrabortyEric N. OuattaraRandall LanierEric O. FreedRanieri BizzarriEric RyndockRanjan K. MohapatraEric TothRanjan RamasamyEric YagerRaphael EberleErich Yukio Tempel NakasuRaphael KochErika FaberRaquel HernandezErin E. SchirtzingerRaquel PortugalErin Elizabeth SchirtzingerRasa Petraityte-BurneikieneErman Salih IstıflıRashed NoorErnest TamboRashid ManzoorErsan AtahanRashid MirErzsébet Horváth-PuhóRashmi SriramEsaú Bojórquez-VelázquezRätsep MatthewEsau JoaoRavi MahalingamEskild PetersenRavinder KumarEsmaeil AmiriRavindra P. VeerannaEsperanza Gomez-LuciaRavins DohareEspinosa Martín Juan CarlosRaymond J. OwensEsteban A. EngelReba KanungoEsteban Hernandez-VargasRebecca ChristoffersonEstéfani García-RíosRebecca DuBoisEstela Paz-ArtalRebecca McLeanEster Ballana GuixRebecca P. WilkesEsther E. Biswas-FissRebecca PoulsonEthan L. MorganRebecca RoseEthel Diane WilliamsonRebecca SkalskyEtienne BrochotRebecca VeenhuisEtienne HerrbachReena Lamichhane KhadkaEugene V. RadchenkoRegiane Maria Tironi De MenezesEugenia BezirtzoglouRegina RoweEugenio RastelliRegis CorreaEui Tae KimRegragui TakiEui-Joon KilReiner StrickEun-Jee OhReinhard ErtlEun-Jin KimReinhild PrangeEunsil ParkReinout Alexander BemÉva ÁyRemi N. CharrelEva HermannRemi PlanesEva NagyRena GorovitsEva Piano MortariRenan Pedra de SouzaEva SpadaRenata DezengriniEva TvrdáRendong FangEva VarallyayRené C OlsthoornEvangelia AntoniouRené LutterEvans StephanieRene Van Der VlugtEvelyn Rivera-ToledoRenée M. Van Der SluisEvelyne ManetRennos FragkoudisEvelyne SchvoererRenu KhasaEvgenia PapakonstantinouRenyi LiuEvgenii GusevReza KhayatEwa BrzozowskaReza Zolfaghari EmamehEwa Jończyk-MatysiakRhoel DinglasanEwa PlantRicardo Augusto DiasEwa TomaszewskaRicardo CardosoEyal KlementRicardo GiordanoEzgi AkdesirRicardo MoratelliFabian B. FahlbuschRicardo ParreiraFabian GiolittiRicardo Soto-RifoFabiana Volpe-ZanuttoRiccardo ScottoFabienne MarcellinRichard A. J. WilliamsFabio Del PieroRichard BowenFabio MonteiroRichard G. HegeleFabio Nascimento Da SilvaRichard J. P. BrownFabio OstanelloRichard J. RollerFabio PossebonRichard KockFabio ScarpaRichard MolenkampFabio ZickerRichard O’HanlonFabrizio FabriziRichard PaleyFahad Al BasirRichard PaulFahad KhanRichard SchlegelFaham KhamesipourRichard StantonFaizal Abdul CareemRichard YanagiharaFan JiaRiina SalupereFan LuoRik de SwartFan ZhuRikio KirisawaFang-Tzy WuRikiya TakeuchiFangzhu ZhaoRineke SteenbergenFanny LegrandRita CordeiroFanos TadesseRitah NakayingaFarah MustafaRitesh MishraFaris Al-LamiRob A. GrutersFaruque AhmadRob EdwardsFasseli CoulibalyRob W. H. RuigrokFatah KashanchiRob WhiteFátima AmaroRobert BurkFatimah Saeed AlhamlanRobert DrillienFatma BerriRobert DudaFatma EldemeryRobert E. LondonFausto MaffiniRobert FlisiakFayna Diaz-San SegundoRobert GarryFederica BrianiRobert GiffordFederica CavalloRobert H. A. CouttsFederica MagagnoliRobert HarrodFederica MonacoRobert J. FischerFederico GarciaRobert J. HawleyFederico GiannittiRobert J. ScarboroughFederico Manuel GiorgiRobert KlamrothFei DengRobert L. HarrisonFei GaoRobert Lopez-AstacioFei KeRobert PosseeFei YuRobert V. StahelinFelicity Jane BurtRobert WatsonFelipe Díaz-GrifferoRobert Y. L. WangFelipe-Andres PiedraRoberta BattistiniFelix BroeckerRoberta BruhnFeng LiRoberta MancusoFeng QuRoberta Maria FachiniFeng RuofeiRoberta MisasiFengbin WangRoberto AlonsoFengchao LangRoberto CodellaFenyong LiuRoberto ScendoniFerdinand Nanfack-MinkeuRoberto Vicente Gozalbo RoviraFerenc JakabRobin WarneFernanda Marcicano BurlandyRocio MontejanoFernando A. OsorioRod RussellFernando BauermannRodolphe BarrangouFernando CardosoRodrigo CarmoFernando EscriuRodrigo Esaki TamuraFernando FerreiraRodrigo GallardoFernando FerreroRodrigo Ligabue-BraunFernando García-ArenalRodrigo RodrigesFernando Gonzalez-CandelasRoger BadíaFernando PonzRoger FrutosFernando RodríguezRoger GrandFernando SpilkiRoger HewsonFerran Acuña-ParésRoger Y. DoddFerran TarrésRokshana ParvinFerruccio BoninoRoland EllingFiammetta ZunicaRoland KlassenFilipe PereiraRoland ZellFilippo LagiRoland ZüstFilomena FioritoRollie J. ClemFilomena FonsecaRomain VolmerFiona FilardoRomana MoutelíkováFisayo OlotuRomina NabieeFlor Ernestina Martinez-EspinosaRomina SalpiniFlor Helene PujolRomulus BrebanFlora de ContoRon DiskinFlorence BuseyneRonald HartyFlorence LarrousRonald L. RabinFlorian KreppelRonald M. IorioFotouh R. MansourRong HaiFrancesca di GiallonardoRonit SaridFrancesca di NunzioRoosevelt Alves Da SilvaFrancesca Marino-MerloRosa de VincenzoFrancesco BonfanteRosa del CampoFrancesco di GennaroRosa María del AngelFrancesco FaggioliRosa Maria N. MarcussoFrancesco FerraraRosane SilvaFrancesco FilippiniRosanna SalviaFrancesco Maria FuscoRosario SabariegosFrancesco MessinaRosemary M. OliveroFrancesco MiraRoss C. LarueFrancesco NazziRossana ScutariFrancesco PapparlardoRossella CacciolaFrancesco SegalaRowe BertrandFrancesco SessaRoxana Mitzayé del CastilloFrancesco StomeoRoy A. HallFrancesc-Xavier Lopez-LabradorRoymans DirkFrancisca SamsingRubaiyea FarrukeeFrancisco ArnalichRuben Cadena-NavaFrancisco EpeldeRuben Hernandez-AlcocebaFrancisco González-ScaranoRuben PérezFrancisco José Nunes AntunesRuben Soto-AcostaFrancisco LlorenteRuchao PengFrancisco MendezRui LuoFrancisco Murilo ZerbiniRuifang WeiFrancisco SobrinoRuoxi ZhangFranck MartinRussell J. DiefenbachFranck TouretRute Gonçalves MatosFranco RopertoRyan J. WinsteadFrancois F. MareeRyan M. PaceFrançois FerrónRyan TroyerFrancois HelleRyan UrakFrancois MaclotRyo OkadaFrançois NiyonsabaRyota TsunekuniFrançois TrotteinRyusei KuwataFrançoise BachelerieSabari Nath NeerukondaFrançois-Loïc CossetSabine Chapuy-RegaudFrank GuentherSabine DiedrichFrank O. AylwardSabitree ShahiFrank OechslinSabri HacıoğluFranklin NobregaSabrina BertinFranklin Wang-ngai ChowSabrina VinzónFranz Xaver HeinzSadaruddin ChacharFraser HillSaddam SaqibFred EyaseSae Hwan LeeFrédéric CoutantSaeid HosseinzadehFrederic Le GalSaeid Najafi FardFrederick BaliraineSaeng-Chuto KepaleeFriederike BachmannSafdar AliFriederike Liesche-StarneckerSaid I. BehiryFriedrich A. GrässerSaif Ur RehmanFumihiro KatoSajjad AhmadFumio ArisakaSakshi AroraFumio SekiSalah HammamiFun-In WangSaleem KamiliFusheng SiSalleh AnnasFu-Shui QuanSally R. RobinsonFuxuan WangSally RobertsGábor KemenesiSalvador HerreroGábor RakhelySalvatore D. BlairGabriel ParraSam WilsonGabriel ReinaSamander KaushikGabriel SantpereSamantha S. SoldanGabriela Chavez-CalvilloSamar TharwatGabriela GoujgoulovaSamara Tatielle Monteiro GomesGabriela Ibáñez-CervantesSami El KhatibGabriela Mellado-SánchezSamir DervisevicGabriela Pastuch-GawolekSamir Mansour Moraes CassebGabriela PopaSamuel A. BerkmanGabriele AnichiniSamuel ConstantGabriele LandoltSamuel DobsonGabriele MadonnaSamuel DominguezGabriele PozzatoSamuel GanGabriella D’EttorreSamuel Ken-En GanGaetano GalloSamuel PushparajGagandeep KaurSamy Mahmoud H. SayedGagandeep SagguSan Ming WangGail E. ReidSanbo QinGail J. Demmler-HarrisonSanchari BhattacharyyaGajanan N. SapkalSandeep KumarGalal MetwallySandeep Kumar SinghGalileu Barbosa CostaSandino Ana MaríaGalina A. ZhouravlevaSandip Ashok SonarGalina KoltsovaSandip N. ChavanGancho PasevSandra BarrosoGang YeSandra Blaise-BoisseauGannon C. K. MakSandra BlomeGareth BradySandra DiederichGarima ArvikarSandra HuérfanoGary MartySandra Laner-PlambergerGary McAuliffeSandra MilićGary McLeanSandra NiendorfGary R. WhittakerSandra OrchardGastón HigueraSandra PirasGaurav VermaSandra SkujaGautam PareekSandra WellerGautam SethiSandrine A. LacourGégout-Petit AnneSandrine CastelainGeMaria Addolorata MariggiòSandrine Valsesia-WittmannGeneroso BevilacquaSandro BindaGeoff HolmSandro GrelliGeoffrey HutinetSandy EldridgeGeoger Eric BlairSang Heui SeoGeok Chin TanSa-nga PattanakitsakulGeorge B. KyeiSanjay Dey George BelovSanjay GoelGeorge CarnellSanjay ReddyGeorge LauSanjaya Kumar SahuGeorge P. LomonossoffSankaran MurugesanGeorge VassilopoulosSanna Maria SillankorvaGeorgios GermanidisSanne TerrynGerald BarrySanta Rasa-DzelzkalejaGerald Fritz SchusserSantiago Mirazo VillarGéraldine PiorkowskiSantiago Tello-MijaresGeraldine TaylorSantosh DhakalGerard F HoyneSantosh GeorgeGerardo Santos-LópezSara El ZahedGerhard DoblerSara Morón-LópezGerhard ThielSara MoutaillerGermán Ernesto MetzSarah BealeGermano CecereSarah E. JacksonGerrit KoopmanSarah L. KempsterGerson KobayashiSarah MulkeyGert C. ScheperSarit AvraniGert Van ZylSarmila TandukarGert VenterSasa RajsicGert ZimmerSascha BrunkeGhadah AlkhaldiSascha TrappGhazi KayaliSascha Y. KupkeGiacomo StroffoliniSasha R. AzarGiada MattiuzzoSastre-Garau XavierGian Luigi RussoSatinder DahiyaGian Paolo AccottoSatoru WatanabeGiancarlo CicoliniSatyavani KaliamurthiGianluca GessoniSaulo D. PassosGianluca RugnaSaurabh ChattopadhyayGianmaria BaldinSaurabh GautamGianni Gori SavelliniSaveez SaffarianGianpiero D’OffiziSaverio PaltrinieriGianpiero ZamperinSaverio ParisiGiantonella PuggioniSayaka MiuraGianvito LanaveSayanta BeraGiau VoSayed Sartaj SohrabGi-Byoung HwangScott B. BieringGilbert Nchongboh ChofongScott P. KenneyGilles DarcisScott ReidGinny MooreScott WeaverGioia BongiornoSe Chang ParkGiordano PulaSead SabanadzovicGiorgio SonninoSean E. LawlerGiorgos P. TsironisSebastian Aguilar PierléGiovambattista de SarroSebastian GisderGiovanni BocciaSebastian Gomez TalquencaGiovanni CattoliSebastian NoeGiovanni FranzoSebastian O. WendelGiovanni La CannaSebastian SchloerGiovanni MariniSébastien A. FeltGiovanni NigroSébastien BoyerGiovanni VillaSebla B. KutluayGipsi Lima-MendezSegio Serrano-VillarGisela SantosSeil KimGisselle N. MedinaSek-Man WongGitana Maria AcetoSelene ZárateGiulia FranzoniSelvakumar SubbianGiulia FreerSelvarani VimalanathanGiulia ScaliaSemir VranicGiuseppe BalistreriSepideh Zununi-vahedGiuseppe BertoniSerafeim C. ChaintoutisGiuseppe LosurdoSerena BernacchiGiuseppe MagliettaSerena BianchiGiuseppe MarruchellaSerge BénichouGiuseppe MurdacaSerge CammilleriGiuseppe NunnariSergei Nikolaevich ShchelkunovGiuseppina La RosaSergey AvdeevGiusi MacalusoSergey NetesovGiusy CardetiSergey V. AlkhovskyGiusy DiellaSergey Y. MorozovGlaucia Paranhos-BaccalàSergey Yu. MorozovGlauco Akelinghton Freire VitielloSergio BabudieriGlenn F. RallSergio CarmonaGleyder Roman-SosaSergio E. RodriguezGloria GriffanteSérgio Oliveira de PaulaGloria Ramirez-NietoSergio PontejoGoedele MaertensSeth PincusGonçalo SilvaSéverine HervéGonzalo de Prat-GaySeverino Jefferson Ribeiro da SilvaGopinath KrishnamoorthySezai YilmazGopinath KrishnasamyShabana BibiGorben PijlmanShafiq Ur RehmanGordana Kenđel JovanovićShafiqul ChowdhuryGordon HamiltonShah Alam KhanGraça PintoShahana MajumderGraham BelshamShahid MansoorGraham BurgessShahzad IrfanGraham HatfullShailly TomarGraham TaylorShambhunath ChoudharyGrant LoganShamir CassimGrazia Anna NiroShanawaz M. GhouseGrazia LicciardelloShangrong FanGrazyna LietzauShannon C. KenneyGregg DeanShannon KenneyGrégory DestrasShannon RoncaGrégory DubourgShantanu GuptaGregory J. FonsecaShaobo WangGregory MelikianShaoli LinGregory SmithShaowei LiGriffith ParksSharad Dattatraya MutalikGrisha PirianovSharat ChandraGrissell TiradoSharifah Syed HassanGrzegorz PiechaSharmi ThorGrzegorz WoźniakowskiSharon Tirosh-LevyGualtiero AlvisiShashank BhargavaGuanghua WangShashank TriphatiGuanghui WuShatavisha DasguptaGuangrong QinShaun PenningtonGuang-Wu ChenShauna KaplanGuangzhao LiShawn BabiukGuanhong WangShawn McLaughlinGuido AntonelliShayne LoubserGuido PapaSheikh Mohammad Fazle AkbarGuido Van MarleSheila TellesGuido VanhamShelley AdamoGuillaume CambrayShelley WatersGuillaume CarissimoShelly RobertsonGuillaume FichesSheng ZhangGuillaume GiraudSheng-Hung ChenGuillermo RisattiSheng-Wen HuangGulam H. SyedShengxi ChenGun TemeeyasenShengzhang DongGunjan ShahSheree YauGunnel HalldenSherif ElkafrawyGuochao WeiShih Jer HsuGuodong RaoShihao LiGuohong CaiShih-Che SueGuohui ZhouShihoko Komine-AizawaGuoqing WangShihua XiangGuoxun WangShihuo LiuGurdeep SinghShih-Yen LoGurudutt N. PendyalaShikha ShrivastavaGustaf E.P. RydellShilpi JainGustavo A. DelhonShin MurakamiGustavo Barcelos BarraShingo NakahataGustavo C. CerqueiraShin-Hong ShiaoGustavo DelhonShinji MakinoGustavo FerminShinji OhnoGuus KochShinji TakamatsuGuus RimmelzwaanShirley Vasconcelos KomninakisGuy LemayShiroma Mangalika HandunnettiGuy RostokerShitao LiGyanendra GongalShivalee DuduskarGyőző L. KajánShixing YangHachung YoonShohei MatsuuraHadeel T. El KassabiShrestha Sinha RayHae-Eun KangShu ShenHafiz-Muhammad-Umer FarooqiShuai LeHaibo WuShuai XiaHaileeyesus AdamuShuang TangHaitham SobhyShuangqi FanHaiyan SunShuchen FengHaiyan YangShufang FanHajime YaegashiShuhong SunHamed Haddad KashaniShuhui SongHamid StajiShumaila HanifHan MeiShumpei WatanabeHan WangShuntai ZhouHana DrahovskáShuofeng YuanHana M. DobrovolnyShuohui LiuHana Mahmutefendić LučinShuping TongHana Van CampenShuzo MatsushitaHande IkitimurShyan-Song ChiouHang FanShyi-Dong YehHang XieSi ChenHangyu ZhouSiamak ZohariHani Ba-AwadhSian FaustiniHan-Jia LinSiddharth SridharHanjun ZhaoSiham FellahiHanna HuebnerSilvana PopescuHanna JöstSilvia BelliniHanne AndersenSilvia BlahakHannelore NeuhauserSilvia FacciniHannes VietzenSilvia García-BujalanceHanns O. LudwigSilvia Gómez-SebastiánHan-Qing YeSílvia I. SardiHans BurgertSilvia PreziusoHans ChengSilvia SauledaHans Georg A. BreitingerSilvia ToricesHans R. GelderblomSilvia WürstleHans ZaaijerSilvija ČerniHansjörg LehnherrSilvina Elena GutiérrezHanyong PengSilvio AntoniakHao LeiSilvio HemmiHao ZhangSimon LyttonHao-Ching WangSimón PobleteHaoqing WangSimon RouxHarald ManggeSimona FiorentiniHarbinder SinghSimona RollaHarikrishnan JayamohanSimone BrogiHarindranath CholletiSimone CavaleraHarini SooryanarainSimone G. RibeiroHarold NoëlSimone GiannecchiniHaroldo ToroSimone VanoniHarrold van den BurgSimons SvirskisHaruka AbeSinu P. JohnHarun AlbayrakSiqing LiuHarvey M. FriedmanSirle SaulHasan AlsaidSisko TauriainenHashimoto KoichiSiva S. PandaHassen S. WolleboSivanesan DakshanamurthyHattaf KhalidSiwaporn BoonyasuppayakornHauck RüdigerSk Mohiuddin ChoudhuryHe SunSlim FouratiHeather WhitakerSmita S. KulkarniHeba A. H. ZaghloulSneha SinghHector Franco de Andrade Jr. Sneha SureshHector MorenoSnjezana Zidovec LepejHéctor QuezadaSo Young YooHector RangelSoham GuptaHeily Carolina Ramirez SantanaSoichiro TakahamaHeinz BurtscherSolomon MainaHeinz‐Peter SchulteissSomya AggarwalHelena JiřincováSong WangHelena Lage FerreiraSonia JangraHelena NejezchlebováSonia MediouniHélène BayssonSonia MorettiHélène CôtéSonia Navas-MartinHelene LiuSonia Vázquez-MorónHelene NorderSonia ZuñigaHelle Bielefeldt-OhmannSonsoles Salto-AlejandreHenk KoppelaarSonthaya TiawsirisupHenner BrinkmannSoon Young PaikHenriette de ValkSophia KooHenry BalfourSophie SmitherHenry L. LevinSoraya MezouarHenry MunyandukiSören LukassenHenry Puerta-GuardoSoufien SghaierHenryk CzosnekSpyridon KintziosHerbert WeissenböckSpyridon StavrouHerman EgberinkSridhar JarugulaHerman FavoreelSrinivas NarasipuraHerman K. EdskesSrinivas Reddy PallerlaHerman SchreuderSriram BalusuHervé PerronStanislav BondarevHichem MoulahoumStanley PerlmanHideki HayashiStavros BashiardesHideki KondoStefan BourgeoisHideo WadaStefan D. MomčilovićHideto FukushiStefan HolzhauserHideto YamadaStefan LüthHidetoshi NomotoStefan PetersHie-Won HannStefan PöhlmannHila ElinavStefan VilcekHiroaki OkamotoStefan WegerHiroaki ShirafujiStefania LauziHirokazu KimuraStefania LeopardiHiroshi BannaiStefania MarsicoHiroshi ShimodaStefanie FruhwürthHiroyasu SakaiStefanie GierHisashi AkiyamaStefano MerlerHisashi IzasaStefano PetriniHo Yin Eric LauStefano RusconiHoang Van ThuanSteffen HauptmannHo-Jong JuStehmann ChristianeHolger LoessnerStephan HamperlHolly HughesStéphan ZientaraHong ZhengStéphane PrietHongli DuStephanie JansenHongliang ChaiStephanie M. LimHongning WangStephanie PoppingHongping JinStephanie SeifertHongping WeiStephen A. RiceHongtao LeiStephen A. SmithHongyan SunStephen AbedonHongzhu CuiStephen Beungtae RyuHonorata KraskiewiczStephen C. HardiesHoseong ChoiStephen D. RyderHosoon ChoiStephen GoffHossam El-BeltagiStephen HsuHoussam AttouiStephen JenningsHoussein AyoubStephen M. LaidlawHsiao-Hsuan KuoStephen MartinHsiao-Huei ChenStephen R. WelchHsin-Yun SunStephen SpatzHua ZhuSteve CarlanHuaji QiuSteve CharetteHuanle LuoSteve DunhamHuanzhou XuSteven BatinovicHuapeng ZhouSteven BradfuteHuaqiang YangSteven D. ZinkHugo A. Barrera-SaldañaSteven F. BakerHugo H. OrtegaSteven HagensHui Yin LimSteven HollowayHuibin LvSteven L. ZeichnerHuichen GuoSteven M. ReedHuifen ZhouSteven M. VallesHuigang ShenSteven ReidHuijun GuoSteven RockmanHui-Min ChungSteven SmithHuldrych GünthardSteven VallesHung-Jen LiuStuart GilmourHung-Yao HoStuart McCluskeyHurng-Yi WangStuart TurvilleHusa PetrSuban K. SahooHuseyin YılmazSubha DasHusni ElbaheshSubhas KhajanchiHye Jin ShinSubodh K. SamratHye Kwon KimSubramaniam SaravananHye Ryoung KimSudhir MorlaHyuk-Joon KwonSudip DuttaHyun Jin KwunSudipti AroraHyung Joo KwonSuiyi TanHyun-Jin ShinSuman AlishettyHyunsuk ShinSuman WattalIan FishSumit MukherjeeIan MolineuxSuncanica Ljubin‐SternakIan TietjenSundaresh ShankarIbrahim El-SayedSundharraman SubramanianIbrahim M. SayedSungchan KimIchiro TakajoSungsu YoukIfeanyi Jude EzeonwumeluSung-Ting ChuangIgnacio RefoyoSunil KarhadkarIgor B. RogozinSunitha JosephIgor JurakSunnie R. ThompsonIgor OscorbinSurekha ShridharIgor RouzineSurendra NegiIgor V. BabkinSuresh GorleIjaz GulSuresh K. TikooIkbal Agah InceSuresh KalathilIlari KuitunenSuresh MittalIlaria Di BartoloSuruchi SinghIldefonso Fernández SalasSusan M. MooreIlias BouzalasSusan MooreIlija BrizićSusan Nadin-DavisIlya TitovSusan PayneIman RahimiSusana B. BravoImbi NurmojaSusana GuixImke SteffenSusana RavassaIndre Kučinskaite-KodzeSusanna Kar Pui LauIndu G. RajapakshaSusannah GoldInger DamonSusanne PfeiferInger NijhofSusetta FinottoIn-Jeong KimSuzanne IngleInna V. DolzhikovaSuzanne L. WarringIoannis A. GiantsisSuzanne M. GilboaIoannis GiantsisSuzanne SandmeyerIoannis KalomenidisSven PischkeIolanda MangoneSvenja Dannewitz ProssedaIoly Kotta-LoizouSvetlana AntonyukIosif MarincuSvjetlana RausIoulia RouzinaSwapna Priya RajarapuIpek KurtbökeSwapnil Ganesh SanmukhIranaia Assunção-MirandaSwathi SangliIrena Duś-IlnickaSwati JainIrene González-DomínguezSwayam PrakashIrene RamosSyed Faraz AhmedIrene RighettoSyed Furqan QadriIrene Sacristán YagüeSyed Hani AbidiIrfan RatherSylvain De BreyneIride Francesca CeresaSylvia Van Den HurkIrina DerkatchSylvie LagayeIrina Isakova-SivakSylwia BudzynskaIrina KiselevaSze-Tsing ZeeIrina Maljkovic BerrySzőke LórántIrina MocanuSzu-Cheng KuoIrina SominskayaSzu-Hao KungIrina TretyakovaTabata Victoria Rosas DiazIris MeditsTadashi MaruyamaIrit DavidsonTadashi WatanabeIsabel García-DorivalTadeusz KaczorowskiIsabella ZanellaTae Yoon LeeIsabelle DarbouxTae-Jin ChoiIshnoor SidhuTae-Ju ChoItzel López-RosasTaeok BaeIuliana StoicaTaha AzadIvan Delgado EncisoTahir FarooqIvan D’orsoTahir RizviIvan NagaevTaís Fukuta CruzIvan NombelaTaisuke IzumiIvan PavlovićTakao IshikawaIvan V. AkhrymukTakao MasudaIvana GrgićTakashi HashimotoIvana LojkićTakashi IrieIvana MaidaTakashi OdaIvanildo P. SousaTakashi OnoderaIvo SirakovTakasuke FukuharaIvone MartinsTakayuki MurataIwona GientkaTakayuki OhshimaIzabela Maurício de RezendeTakele ArgawIzabela Mauricio RezendeTakeo KuwataIzabela RaganTakeya TsutsumiJ. Stephen LodmellTakeyuki ShimizuJacco BoonTakuya FukushimaJacek BoguckiTakuya TadaJacek KuźmakTal LevinsonJack HearnTamaki OkabayashiJacob HochmanTamás LetohaJacomine K. Krijnse-ĹockerTami CourseyJacquelina WoodsTamiko HisanagaJacqueline KingTamiru AlkieJacques FantiniTamryn MarsbergJacques Le PenduTanel PungaJacques TheronTanja GerlzaJagannadha SastryTao ChenJagdish KhubchandaniTao DengJai Singh PatelTao LiJaime A. Cardona-OspinaTao YunJakob McBroomeTar Choon AwJakob TrimpertTara M. StruttJakub KubackiTarek H. El-MetwallyJames B. StantonTarja SironenJames BinleyTaro IkegamiJames BrienTaro KamigakiJames EagleshamTasneem MotiwalaJames FoxTassanee SilawanJames J. KohlerTatiana BetakovaJames LogueTatiana V. KomarovaJames NettlesTatiane AssoneJames Romero-MastersTatjana Avšič-ŽupancJames ZhuTatjana Vilibić-ČavlekJamil SaadTatsufumi UsuiJamirah NazziwaTatsunori NakanoJamshid RazmyarTatsuo KandaJan A. RuzickaTatsuo ShiodaJan HellertTatsuo SuzutaniJan MrazekTatyana A. TimofeevaJan PaczesnyTatyana IlyichevaJan R. T. van WeeringTavis K. AndersonJan RichterTea GlontiJan WeberTelung PanJana TomáškováTengchuan JinJanaina FernandesTerenzio CosioJane C. BurnsTeresa CapellJane L. KoTeresa de los SantosJane OakeyTeresa LettiniJanet DalyTeresa SantantonioJanet NaleTerje DoklandJanet TateTero AholaJanhavi P. NatekarTerry A. KleinJanine MichelTerry BowlinJanovec VaclavTetiana ShevchenkoJansons JurisTetsuo KoshizukaJaquelin P. DudleyTetsuro IkegamiJaqueline Mendes de OliveiraThais GuimaraesJared BullardThanuja Thekke-VeetilJared C. RoachThavasimuthu CitarasuJared MayThawornchai LimjindapornJaroslav PejchalTheam Soon LimJarosław PinkasTheo SklaviadisJarosław WaloryTheocharis TsoleridisJarrod J. MousaTheodore MeyersJaru-Ampornpan PeeraTheresa Li-Yun ChangJasmina LuczoThibault MesplèdeJasna RakonjacThomas ChambersJason BodilyThomas George EvansJason C. HuangThomas GrunwaldJason ClarkThomas HansenJason E. ShoemakerThomas HarrerJason MercerThomas HoenenJason MitchellThomas IftnerJavad AlizargarThomas K. HironJavier A. JaimesThomas KlimkaitJavier Castillo-OlivaresThomas KristieJawhar GharbiThomas M. ChappellJay AnandThomas MichielsJay BrownThomas MurookaJay KrishnanThomas Sicheritz-PonténJay R. RadkeThomas SpranghersJay TrivediThomas StammingerJayasri DassharmaThomas TuJayeshbhai ChaudhariThomas YuillJean Carlos BettoniThorsten G. MüllerJean Christophe PaillartTiago Souza SallesJean Claude PerezTian DebinJean François ÉtardTian WangJean HarryTianshu XiaoJean K. MilletTihamer MolnarJean KanitakisTill AndreasJean Luc BaillyTill RümenapfJean MukherjeeTimm HarderJean Philippe KevorkianTimo H. VesikariJean Pierre KabueTimo HautalaJean VanderpasTimothy G. BergerJean-Francois Lauzon-JosetTimothy J. ScaseJean-Louis BayartTimothy John MahonyJean-Luc MurkTimothy P. AlgeoJeanmarie VerchotTimothy P. NewsomeJean-Michel DrezenTimothy ScaseJeanne Brugère-PicouxTina O’GradyJean-Pierre AllainTingyu ChengJean-Pierre FrossardTirumala Kumar ChowdaryJean-pierre MolesTiziana ZottiJeesun ChunTobias RomeykeJeff WiluszTobias SchmidtJeffery DeStefanoTodd BradleyJeffery L. MeierTohru SuzukiJeffrey A. JohnsonTom HobmanJeffrey CornuaultTomas CantoJeffrey SeowTomás G. VillaJelena PrpicTomasz A. StenzelJelke FrosTomasz StompórJennifer A. CorcoranTomasz TokarekJennifer F. MoffatTomaž Mark ZorecJennifer HeadTomohiko SadaokaJennifer L. CannonTomohiro IshikawaJennifer MahonyTomoko HanawaJennifer TotonchyTomoko KobayashiJenny HessonTomomasa MatsuyamaJeong HeoTomomi TakanoJeong Yoon LeeTomoo SatoJeong-An GimTomoyoshi DokiJeong-Min KimTomoyuki MurakamiJérémie GrasTomozumi ImamichiJérémie PrévostTong ZhangJeremy P. WoodTongling ShanJeremy ThompsonToni A. ChapmanJeremy V. CampToni TodorovskiJerome Delamare-DebouttevilleTonya L. TaylorJérôme GouttenoireTorsten SeuberlichJérôme LeGoffToru TakahashiJerónimo González-BernalToru TakimotoJesse ArbuckleToshana Lauria FosterJesse DeereToshi NagataJessica A. CanterToshihiro KitaJessica A. PlanteToshihiro NagamineJessica BelserToshikazu SuzukiJessica CoertseToshinungla AoJessica R. HarmonToyoyuki OseJesús Adrián LópezTraian DumitrașcuJesus HernandezTridib RajkhowaJesús L. RomaldeTrifan Anca-VictoritaJesús Navas-CastilloTrudi O’NeillJesus Omar Muñoz BelloTrudy G. MorrisonJesús Rodríguez-DíazTsuge MasatakaJhon Castaño-OsorioTsuguru HayashiJia HeTsung-Hsien ChangJia LiuTsung-Hui HuJiachen HuangTsutomu MurakamiJiae KimTsutomu OmatsuJiafen HuTsuyoshi ShiraiJiagang TuTuan L. PhanJiahai LuTuba Çiğdem OğuzoğluJiahui DingTudor BorzaJialing BaoTudor Lucian PopJian XuTugba Taskin TokJian YangTuli MukhopadhyayJian YeTulio Machado FumianJian ZhangTyler RadnieckiJianchao ZhangTyng Yuan JangJiang ZhongTzu-Chieh TangJianghong LiTzu-Chuan HoJianhao CaoUdhaya Kumar S. Jianhua WangUgo Lionel MoensJianing FuUlf PetterssonJianming QiuUlises Gomez-PinedoJianming WuUlrich DesselbergerJianxuan WuUma S. SajjanJianzhu LiuUmberto BasileJiarong GuoUmberto MoliniJiaxin LingUnai Perez-SautuJiayi WangUrmi RoyJiban Kumar KunduUrška KuharJie CuiUtpal TatuJie LiV. Gregory ChincharJieke JiangVaibahv JainJieqing ZhuValentin A. SemenovJieshi YuValentina CirkovicJilong ChenValentina Dell’OsteJim F. KaufmanValentina MazzottaJim M Pr DunwellValentina SvicherJin WeiValentina TerioJin Woo JunValentina Virginia EbaniJing CaoValeria CagnoJing JinValeria CentoJing ShangValeria GhisettiJing XingValeria ListortiJingen ZhuValeria ManganelliJingguang WeiValeria PietropaoloJing-Houng WangValeria RondinoneJingyou YuValérie ChoumetJinhong ChangValerie Le SageJinlin LiValerija GromaJinping ChenValerio FulciJinsong LiuValerio LeoniJinwei ZhangValesca AnschauJinyang ZhangValtýr Stefánsson ThorsJinzhi NiuVamsee MallajosyulaJiong ChenVanessa D. CostaJiong ZhouVanessa de PaceJiří DoškařVanessa HerderJiří HejnarVanessa Meier-StephensonJitender P. DubeyVanja KaliternaJitendra KumarVanvimon SaksmerpromeJitka MucksováVarsha PotdarJiufeng WangVasileios PapatsirosJiyan MaVassilios A. PaparizosJoachim DennerVeasna DuongJoachim Ewald KühnVeerasak PunyapornwithayaJoan L. KenneyVeerendra SharmaJoana VitalléVeerle MsimangJo-Ann LeongVeerle VanlerbergheJoanna SajewiczVeijo HukkanenJoanna Sztuba-SolinskaVelasco CimicaJoanne HewittVelmurugan BalaramanJoanne HiebertVenkat YedavalliJoão MesquitaVenkatraman SiddharthanJoão Renato Rebello PinhoVenkatramana KrishnaJoaquim CarrerasVeronica C. HoadJob HarenbergVerónica Chico GrasJochen WettengelVeronica Di CristanzianoJodi HedgesVeronica Mata-HaroJody K. MaysVerónica TrunigerJoe A. FralickVéronique Avettand-FenoelJoe ChihadeVesa HytönenJoe JamesVesna MilicevicJoe MymrykViacheslav S. SmirnovJoel BainesViatcheslav MartemyanovJoel RovnakVicente MonederoJoel V. ChuaVicente SorianoJoerg-Matthias PollokVicki L. Traina-DorgeJohan NordgrenVictor HuberJohanna E. FraserVíctor M. GonzálezJohannes BlümelVictor MatheuJohn A. Albert St. CyrVictoria A MeliopoulosJohn C. TrefryVictoria IndenbaumJohn David BeckhamVictoria JenningsJohn DunnVictoria K. BaxterJohn J. HalperinVictoria Pando-RoblesJohn LambertVictoria WahlJohn Lok Man LawVida Van StadenJohn M. GonzalezViet Loan Dao ThiJohn Mackay SøftelandVijay ReddyJohn McCauleyVijay Singh GondilJohn MoldovanVikram MisraJohn RobertsVincent ParissiJohn TavisVincenza GianfrediJohn TaylorVincenzo Di Marco Lo PrestiJohn W. MellorsVincenzo QuagliarielloJohnatan Torres-TorresVinod BalasubramaniamJohn-Sebastian EdenVipin HallanJojanneke van SummerenVirginia M. StoneJolanta KiełpińskaVirginia ProttoJolene CarlsonVirginie DoceulJon SinVirginie MortierJonas HerzbergVirginija Cvirkaite-KrupovicJonas Nascimento CondeVishal NegiJonatas S. AbrahaoVita GolubovskayaJonathan FeelemyerVitaly GanusovJonathan GuitoVitaly V. KushnirovJonathan RaynerVito MartellaJonathan RichardVittorio SambriJonathan RunstadlerVittorio SarcheseJonelle MattiacioVivekanandan PerumalJong-Soo LeeVivian K. O’DonnellJoon Kee LeeViviana ClavijoJordi ArgilaguetViviana ManzulliJordi Serra-CoboViviana ParreñoJorge C. G. BlancoVladimir BaklaushevJorge CassebVladimir E. FrankevichJorge Reyes-del ValleVladimir Ivanovich PotkinJorlan FernandesVladimir MuronetzJorma HinkulaVladimir SavicJort Jort VellingaVladimir StevanovićJos M. B. M. Van der VossenVladimir V. KovalJose A. Regla-NavaVladimir V. ZarubaevJosé AlcamiVojko KanicJosé Alejandro BohórquezVojtěch ŠrollerJose Alejandro Martinez-IbarraVolker LohmannJosé AlmendralVolker NickeleitJosé BlancoVolker SchmidtJosé Bustos-ArriagaWagner QuintilioJosé Carlos de MagalhãesWaldemar WagnerJosé CuevasWalid AbdelwahabJosé Francisco IslasWalid AzabJosé González-SantamaríaWallace CasacaJosé Guadalupe Soñanez-OrganisWalter AtwoodJosé Jiram Torres-RuizWalter DoerflerJosé Joaquín CerónWalther MothesJosé Luis AffranchinoWang ShengJosé Luis Rodríguez GutiérrezWang ZhengJosé Manuel Jaramillo OrtizWanpen ChaicumpaJose MateusWayne GrayJosé Miguel Azevedo-PereiraWayne ThomasJosé P. Cerón-CarrascoWei LuoJosé Ramos VivasWei TengJose Sanchez-BetancourtWei WangJoseph A. WestrichWei XiaoJoseph C. KidneyWei XuJoseph E. EbingerWei ZouJoseph HeffronWei-Fan HsuJoseph KendraWeiguang ZengJoseph LubegaWeihong QiJoseph MarcotrigianoWeihua HuangJoseph Sriyal Malik PeirisWei-June ChenJosh ColstonWeili KongJoshua FreemanWeiming OuyangJoshua J. AnzingerWeisheng CaoJoshua Oluoch AmimoWeiwei ZengJosip A. BorovacWeiwei ZhangJosué Díaz-DelgadoWenbao QiJovana BozicWen-Chi SuJovana MilicWendy MauryJovita Fernández-PineroWenhui HuJózef J. BujarskiWenjing GuoJózsef KónyaWenli ZhangJózsef PrechlWen-Quan ZouJózsef TőzsérWenshih TsaiJuan A. BonachelaWen-Wu LinJuan Antonio GarciaWenying LuJuan BarcenaWerner J. D. OuwendijkJuan Bautista de SanctisWesley Luzetti FotoranJuan Carlos NavarroWhitney QuallsJuan DegiuseppeWildriss ViranaickenJuan DuWilliam BrittJuan E. LudertWilliam L. IrvingJuan FontanaWilliam SwitzerJuan José FernándezWilliam T. Gunning IIIJuan José López-MoyaWilliam W. DavisJuan Pablo JaworskiWilliam WilsonJuan Pablo TosarWillian P. PaimJuan PerillaWilly M. BogersJuanjie TangWilna VoosloJu-Chien ChengWilson Lewis MandalaJudit J. PénzesWim van BortelJudit PenzesWojciech DrygasJudith M. HübschenWojciech RozekJudy BeelerWolfgang BeyerJue LiuWolfgang FischerJuhee AhnWolfram GerlichJukka T. MustonenWon-Il KimJulia DinaWuhan XiaoJulia Rebecca PortWuxiang GuanJulia RidpathXi ZhouJulian HillyerXianfu WuJulian Ruiz-SaenzXiang RenJulian TangXiangjie SunJuliana Reis CortinesXiangjun DuJuliane RöderXiangru ChenJulianne GroseXiangyang LiJulie BoucauXiaodong LianJulie FoxXiaofeng WangJulie ThomasXiaohong ZhouJulien AndréaniXiaohui ZhangJulien BéthuneXiaoke XuJulien CarvelliXiaoling ZhouJulien MéladeXiaoming ZhangJulien PomponXiaonan ZhangJuliet V. SpencerXiaotian TangJulio PascualXiaoting ZhangJulio Retamales LaraXiaoyu NiuJulio Rodriguez-AndresXiaozhen LiangJulio SoteloXiaozhou MaJulio ToralesXimena Olarte-CastilloJulio Vicente Figueroa-MillánXin LiJumari SteynXin WangJun AriiXin YangJun HangXIng YangJun JiangXingbo XuJun KuritaXinghong DaiJun LiXinglou YangJun PengXinlei LiJung-Eun ParkXinling WangJung-Hoon KwonXinran LiuJunichi KawadaXintao HuJunichi YoshidaXinyue ChangJun-ichirou YasunagaXinzhuan SuJunko S. TakeuchiXionglin FanJunko TanakaXiulian SunJunmei ZhangXiuqin WangJunmin LiXiuqiong BiJunping YuXizhi GuoJunwei LiXuanyi WangJunyan HanXue Zhi ZhaoJürg NüeschXuefeng LiuJurica Jug-DujakovićXuefeng YuanJustin A. RobyXueping ZhouJustin H. J. NgXuesen ZhaoJustin KurianXuexian YangJustin PennerXueyong ZhuJustin PollaraXuhua XiaJustin R. ClarkXulin ChenJustin V. McCarthyXupeng HongJustine CharonXusheng QiuJustyna BroniarczykXuyong LiJustyna DunajYael Fridmann-SirkisJustyna GołębiewskaYael GozlanJutta EichlerYan SunJutte J. C. de VriesYan YuanJu-Yeon YoonYanan LiuJyunsuke ShiraiYang HuKachun ChongYang QiuKadriye CaglayanYang WangKai Alexander KroppYanink Caro-VegaKai QinYanmin WanKai WangYanni SunKai XuYanni XiaoKai ZhengYannick BlanchardKaicheng WangYannick BrüggemannKaixin LiangYannis DrossinosKajal BiswasYanping TianKalyani PuttyYaoqing ChenKamalakar ChatlaYaosheng ChenKamaleldin SaidYash GuptaKamalendra SinghYashavanth Shaan LakshmanappaKamalika MojumdarYasir WaheedKame Alberto Galan-HuertaYassmin MoatasimKamil F. KhafizovYasuhiro YasutomiKang MaoYasuhito IshigakiKang NingYasuhito TanakaKanok PreativatanyouYasutsugu SuzukiKaori TerasakiYau-Heiu HsuKar MuthumaniYe ChenKarel KostevYe WangKaren A. AlroyYe-Fan HuKaren BeemonYegor S. VassetzkyKaren BohmwaldYen-Hung ChowKaren KlyczekYi LiKaren LaurieYi-Cheng ChenKaren P. AckerYifei LiaoKaren SeiterYifeng JiangKarim EssaniYihang ShenKarina A. ToledoYin LiKarine BarralYing FengKarine DubeYing KongKarl BoehmeYing WangKarl Kai McKinstryYing ZhangKarl MungerYingfeng LeiKarl RobinsonYingjie SunKarla L.H. FeijsYingying CongKarla PassalacquaYingying GuoKarmun ChooiYiping LiKarolina AkinosoglouYiu Wing KamKarolina TarasiukYixuan MaKasha P. SinghYohan JangKatalin SalánkiYoke Fun ChanKatarina BačnikYolanda RevillaKatarina HancevicYong PoovorawanKatarzyna Domańska-BlicharzYong-Ai XiongKatarzyna Otulak-KoziełYongcai LiKatarzyna TrzmielYong-chan KimKate Morris MaryYongfen XuKate WoodworthYong-Hui ZhengKaterina StratiYongjun SuiKatharine E. MagorYongkui LiKatherine Laiton-DonatoYongli XiaoKatherine SiddleYongming SangKatherine V. WilliamsYongqin LiuKathleen Boris-LawrieYong-Suk JangKathleen HefferonYongtang ZhengKathrin HanleyYongzhen LiuKatie TormanenYoon-Seok ChungKatja LammensYoshifumi IwamaruKatrin HartmannYoshihiko YanoKatsuhiro KomaseYoshihiro SakodaKatsunori OkazakiYoshihisa HanayamaKaveesha WijesingheYoshihisa YamanoKaw Bing ChuaYoshikazu Honda-OkuboKawang LiYoshimi Enose-AkahataKayla SprengerYoshinao KuboKayvan EtebariYoshiyuki SuzukiKazuaki ImaiYosuke MatsushitaKazuaki MondeYotam Bar-OnKazuaki YoshimuneYoung-Jun ParkKazuhiro IshibashiYoung-Keol ChoKazumi NakanoYoung-kyo SeoKazumoto MurataYoung-Suk LimKazuo NakamichiYou-Wen HeKazushi MotomuraYper HallKefang LiuYu ChenKegong TianYu JiemeiKei MiyakawaYu NakagamaKei YuasaYu XiangKeiji UedaYuan-Hung WangKeisuke IshizawaYuan-Ti LeeKeita WagatsumaYuanyuan LiKeith ChappellYu-Chan ChaoKeith E. ShearwinYuchen XiaKeith LeppardYu-Chung ChangKeith RobertsonYue QinKeittisak SuwanYuefei JinKejun GuoYuejiao XianKelli L. BarrYuekun LangKelly S. HarrisonYuetsu TanakaKen S. RosenthalYue-Wen ChenKen StedmanYuhao LiKenji S. NakaharaYu-Huan TsaiKenneth A. StaplefordYuji TomaruKensuke HirasawaYuki HagaKenta OkamotoYuki OtsukaKenta TeruyaYuko MorikawaKentaro SekineYulia VakulenkoKentaro TohmaYumeng YanKentaro YamadaYun ShiKentaro YoshiokaYun WangKerstin KrügerYun ZhuKeun Hwa LeeYu-Na LeeKevin BohannonYunlong CaoKevin D. PavelkoYu-Nong GongKevin FonsecaYuntao LiuKevin GauthierYunxue GuoKevin John SelvaYuriy DavidyukKevin M. CoombsYuriy Fedorovich DryginKevin SokoloskiYury ChernoffKevin TsaiYusuke KatoKevin YehlYusuke MatsumotoKevin ZwezdarykYuta ShiroganeKhalid M. DousaYutaka TagayaKhalid MuhammadYutaro NeriyaKhalid RazaYuwen LuKhalid TimaniYves GaudinKhalil AhmedYves MelyKhalil EttayebiYvonne DrechslerKhatkar PoojaYvonne SuKhristine Joy C. AntiguaZahra KadriKi Hong KimŻaneta Kimber-TrojnarKi Hyun NamZarębska-Michaluk DorotaKi Kwang OhZdenék KejíkKiattawee ChoowongkomonZedong LiKieu The Loan TrinhZejun LiKilian SchoberZekun MuKim BlasdellZenon LukaszewskiKim HalpinZeyang ZhouKim SteegenZezhong LiuKimberly A. Bishop-LillyZhangyong NingKimberly M. ChristianZhanqi DongKimihito ItoZhe ZhaoKimmo VirtanevaZhen WangKin Kui LaiZheng ZhangKin-Hang KokZhengli ShiKiramage ChathurangaZhenglun LiangKiran KondabagilZhenhua ZhengKirill SharshovZhenjian HeKishan Kumar NyatiZhenlong LiuKiyosu TaniguchiZhenyu ZhangKiyotaka ShibaZhi YangKlaudija ViškovićZhicheng CuiKlaus HedmanZhike FengKlaus PetryZhipeng YanKlaus StrebelZhiqiang QinKo KoZhiqiang WuKobporn BoonnakZhitao QiKoen BartholomeeusenZhiwei CaoKoenraad van DoorslaerZhiyong MaKoh FujinagaZhiyou DuKohji MoriishiZhong HuangKohji NoguchiZhongtian LiuKoki TaniguchiZhongwu ZhouKondabattula GaneshZhou JinKonigheim KonigheimZhou XingKonstantin Gus KousoulasZia Ur RehmanKonstantin M. J. SparrerZichao MiaoKonstantin SharovZifu ZhongKoray ErgunayZijian ZhangKore SchlottauZijun WangKosuke MurakamiZiling QiaoKosuke OkuyaZiqiang ChengKouichi KitamuraZiwei YeKoureas MichalisZiwen WangKousho WakeZiying YanKow Tong ChenZizheng ZhengKowit HengphasatpornZoltán BánkiKrishna ShrinivasZoltán DeimKrishnaswamy Gopalan TirumurugaanZoltan VajoKristen A. McLaurinZon Weng LaiKrit PongpirulZongdi FengKrithika RajagopalanZongjie WangKrzysztof ŚmietankaZsolt FeketeKrzysztof TrederZsolt RuzsicsKseniya TuchynskayaZsolt TothKuancheng LiuZulqarnain BalochKubilay YıldırımZunlong KeKulbhushan Sharma



